# An Exploratory Study of Six-Month Niacinamide Supplementation on Macular Structure and Electrophysiology in Primary Open-Angle Glaucoma

**DOI:** 10.3390/vision10010007

**Published:** 2026-01-28

**Authors:** Constantin Alin Nicola, Maria Cristina Marinescu, Cristina Alexandrescu, Anne Marie Firan, Walid Alyamani, Mihaela Simona Naidin, Radu Constantin Ciuluvica, Radu Antoniu Patrascu, Anca Maria Capraru, Adina Turcu-Stiolica

**Affiliations:** 1 Doctoral School, University of Medicine and Pharmacy of Craiova, 200349 Craiova, Romaniaanca.mitran@umfcv.ro (A.M.C.); 2 Discipline of Medical Physiology, Faculty of Medicine, Carol Davila University of Medicine and Pharmacy, 020021 Bucharest, Romania; 3 Department of Ophthalmology, Faculty of Medicine, Carol Davila University of Medicine and Pharmacy, 050474 Bucharest, Romania; 4 Barnsley Hospital NHS Foundation Trust, Barnsley S75 2EP, UK; 5 Department of Pharmaceutical Marketing and Management, Faculty of Pharmacy, University of Medicine and Pharmacy of Craiova, 200349 Craiova, Romania; 6 Discipline of Anatomy, Faculty of Dentistry, Carol Davila University of Medicine and Pharmacy, 020021 Bucharest, Romania; 7 Department of Health Economics and Outcomes Research, Faculty of Medicine, Iuliu Haţieganu University of Medicine and Pharmacy, 400012 Cluj-Napoca, Romania

**Keywords:** glaucoma, visual field, optical coherence tomography, visually evoked potentials, vitamin B3, niacinamide, nicotinamide

## Abstract

Background and Objectives: Primary open-angle glaucoma (POAG) is one of the leading ocular diseases leading to irreversible blindness and is often asymptomatic until advanced cases. While intraocular pressure reduction remains the cornerstone of treatment, neuroprotective strategies targeting retinal ganglion cell metabolism are actively investigated. Niacinamide (nicotinamide, vitamin B3), a precursor of NAD+, has shown neuroprotective potential in preclinical models. This exploratory study evaluated the short-term functional, structural, and electrophysiological effects of oral niacinamide supplementation in POAG. Materials and Methods: In this interventional study, patients with POAG received oral niacinamide 500 mg daily for six months. Visual field (VF) global and localized sensitivity (Mean Deviation [MD], Pattern Standard Deviation [PSD]), Optic Coherence Tomography (OCT)-derived peripapillary retinal nerve fiber layer (RNFL) and macular ganglion cell complex (GCC), and Visual evoked potentials (VEP) latency parameters (P2 1.4 Hz, P100 1°, and P100 15′) were assessed at baseline and at six months. Because both eyes from some participants were included, primary longitudinal inference was based on clustered analyses using generalized estimating equations and linear mixed-effects models to account for inter-eye correlation. Eye-level paired analyses were used for exploratory comparison. Change–change relationships across modalities were explored using Spearman correlation. Results: After accounting for inter-eye correlation, no statistically significant change in MD was detected (mean ΔMD +0.43 dB; GEE *p* = 0.099; LME *p* = 0.101), and PSD remained stable. RNFL thickness showed a small decrease (−1.26 µm; GEE *p* = 0.046), while GCC did not change significantly. VEP P100 latencies remained stable, whereas P2 latency showed a small increase (+3.9 ms; GEE *p* = 0.039). Correlation analysis revealed a moderate association between changes in GCC and MD (ρ = 0.44), suggesting concordance between macular structural stability and global visual field performance. Conclusions: When inter-eye correlation is appropriately accounted for, six months of niacinamide supplementation in POAG is associated with overall functional, structural, and electrophysiological stability, without evidence of clinically meaningful improvement or progression. These findings support short-term safety and highlight the importance of clustered analytical approaches and macular-centered biomarkers in future glaucoma neuroprotection trials.

## 1. Introduction

Primary glaucoma is one of the most common ocular pathologies, with a high risk of irreversible blindness: it is estimated that the lifetime risk of blindness is 10% in primary open-angle glaucoma (POAG) and 25% in primary angle-closure glaucoma, pathologies which present the common refractive errors, myopia and hyperopia, as important risk factors [1,2]. A recent meta-analysis estimates that the pooled prevalence of glaucoma in European populations is 2.60% [3]. More recently, the estimated prevalence of glaucoma is 2.99% in Europe, with a higher prevalence in male and older patients. More than half of the studied cases were previously undiagnosed, and worryingly, over 80% of undiagnosed cases were in young people under 55 years old [4].

Several mechanisms are incriminated in the debut and progression of glaucoma, with main hypotheses being the mechanic and ischemic ones, and currently, the only therapeutic mechanism in glaucoma is the control of intraocular pressure (IOP) by medically or surgically modulating the production and drainage of aqueous humor [5]. However, recently, more and more research is dedicated to the neuroprotection mechanism—which does not involve decreasing IOP in order to limit the damage to retinal ganglion cells (RGCs) and nerve fiber layer (RNFL) but directly maintaining the health of RGCs and avoiding their death (which constitutes the glaucomatous neuropathy) [6]. One potential neuroprotective strategy is by modulating mitochondrial dysfunction, known to be involved in axonal degeneration in glaucoma [5].

Niacinamide and nicotinamide refer to the same compound, the amide form of vitamin B3. In the cellular metabolism, niacinamide acts as a precursor to nicotinamide adenine dinucleotide (NAD+), which is involved in a series of essential mitochondrial functions: it acts as a coenzyme in redox reactions (NAD+ can accept a H+ ion and reduce to NADH, and thus be involved in pathways such as glycolysis, glutaminolysis, and fatty acid oxidation) and as a coenzyme/substrate in non-redox reactions, involving sirtuins and Poly(ADP-ribose) polymerases (PARPs) in processes involved in DNA repair, intracellular calcium signaling, and gene expression [7,8,9,10].

Niacinamide has been shown in preclinical animal studies to offer potential benefit: it was shown that NAD levels decrease in the retina, rendering RGCs susceptible to IOP-driven degeneration. By either supplementing orally or by gene therapy to induce intrinsic NAD synthesis, studies in animals have shown niacinamide to protect against RGC death in glaucoma models [11]. Further, initial human trials are underway and suggest potential benefit, and a meta-analysis showed a lower dietary intake of niacin in glaucoma patients and a 33–37% lower likelihood of glaucoma in a high-intake group [12,13,14].

There are several paraclinical tools to support the diagnosis and follow-up of glaucoma: the visual field (VF) is essential, together with Optic Coherence Tomography (OCT), and a valuable addition is the Visually Evoked Potentials investigation (VEPs). In performing a VF, the light sensitivity of the retina is measured, usually by an automatic method (static perimetry), in the central points of the retina (in glaucoma, the central 24 degrees around the fovea, but testing 30 or 10 degrees is also useful) [15]. Typical patterns of visual field dysfunction in glaucoma include a nasal step defect (the earliest in the disease’s progression), paracentral and arcuate scotomas, somewhat more commonly in the superior hemifield compared to the inferior one, or diffuse visual field loss (though rarer and more non-specific) [16]. OCT is a non-invasive imaging technique using low-coherence interferometry to measure the thickness of the retinal nerve fiber layer (RNFL) around the optic nerve head (ONH), together with the ganglion cell complex at the macular level (GCC), which includes RNFL, the ganglion cell layer (GCL), and the inner plexiform layer (IPL)—corresponding to the body, axon and dendrites of the ganglion cell—which is the epicenter of glaucomatous damage [17]. Last but not least, electrophysiological testing may bring valuable data for a glaucoma patient; specifically, VEP may screen for RNFL loss, and may support diagnosis before VF defects. In this method, the occipital cortex’s electrical signal is measured as a response to light stimuli [18].

In this open-label, single-arm exploratory study, we have followed the evolution of POAG patients following B3 supplementation in terms of objective perimetric, tomographic, and functional measures, reporting a predefined 6-month interim analysis designed to assess the feasibility, short-term safety, and multimodal responses associated with niacinamide supplementation, without implying treatment efficacy. We aim to generate hypotheses and estimate variance for a future randomized controlled trial (RCT).

## 2. Materials and Methods

### 2.1. Patients

This was an interventional study, with a non-randomized allocation. Given the exploratory, single-arm design, a formal power calculation was not performed. The study group constituted patients who presented consecutively to a private ophthalmology practice in Bucharest, Romania, between March 2023 and March 2024. The study follows the Declaration of Helsinki and was approved by the Ethics Committee of the University of Medicine and Pharmacy of Craiova, Romania (no. 50/10.02.2023). All patients offered written informed consent. The study is registered at ClinicalTrials.gov with ID NCT07007260.

The inclusion criteria were the prior diagnosis of primary open-angle glaucoma (POAG), with pressure control maintained either surgically or with topical treatment. The exclusion criteria were as follows:

- Low testing compliance (high frequency of errors on VF examination);
- Pregnancy;
- Very advanced glaucoma (Blindness);
- Other significant ocular pathology (advanced cataract, degenerative myopia, keratoconus and other corneal ectasia, amblyopia, vitreoretinal pathology, ocular inflammation, or significant sequelae of trauma or inflammation).

The flow diagram of including the patients in the cohort study was presented in our previous article [19]. The subjects of the study underwent an ophthalmological consultation, which involved measuring the best corrected distance visual acuity (expressed in the logMAR scale), a biomicroscopical examination of the anterior and posterior segment (from which the cup-to-disc ratio, or C/D, was extracted), and an IOP measurement (using the I-Care tonometry device, Icare Finland Oy, Espoo, Finland). Data analyzed also included the disease duration in years and the number of antiglaucomatous molecules administered topically daily. All cases were classified following the Bascom Palmer GSS criteria (VF criteria):

- Stage 1 (early glaucoma): MD over −6.00 dB;
- Stage 2 (moderate glaucoma): MD over −12.00 dB;
- Stage 3 (advanced glaucoma): MD over −20.00 dB;
- Stage 4 (severe glaucoma): MD −20.01 dB or worse [20].

Further, patients underwent the following examinations, all performed by the same operator (M.C.M.) and the same device was used for all patients:

- A Humphrey Field Analyzer (HFA II, Carl Zeiss Meditec, Inc., Dublin, CA, USA) was used to measure the 24-2 Computerized Visual Field and report the Mean Deviation (MD) and Pattern Standard Deviation (PSD);
- Optical coherence tomography (OCT—Zeiss Cirrus HD-OCT 5000, Carl Zeiss Meditec, Inc., Dublin, CA, USA) of the macula and optic nerve head;
  ○ Ganglion Cell OU Analysis: Macular Cube 512 × 128—in order to determine average ganglion cell complex thickness (GCC);
  ○ ONH and RNFL OU Analysis: Optic Disc Cube 200 × 200—in order to determine the average Retinal nerve fiber layer thickness (RNFL).
- Visually evoked potential (VEP) (RETI-port/scan 21, Roland Consult Stasche & Finger GmbH, Brandenburg an der Havel, Germany) included the following:
  ○ Flash VEP in order to determine P2 waveform latency;
  ○ Pattern-reversal VEP (PRVEP) in order to determine the P100 waveform latency for larger, 1.0 degree (60 min arc)-sized checkerboard stimuli;
  ○ Pattern-reversal VEP (PRVEP) in order to determine the P100 waveform latency for smaller, 15 min arc-sized checkerboard stimuli.

To minimize the learning effect, all patients included in the study had performed, throughout their glaucoma follow-up, at least 3 visual fields before being included in the T0 phase of the study. Visual fields were considered reliable if fixation losses were ≤20%, false-positive responses ≤ 15%, and false-negative responses ≤ 25%. Tests not meeting these criteria were excluded from analysis.

OCT scans were eliminated if they presented a signal strength of 6 or less, if they presented any artifacts (motion or blinking), or if the image presented decentration or segmentation errors. Manual correction was not performed.

The VEP measurement followed the ISCEV standard for clinical visual-evoked potentials [21]. The stimuli were pattern reversal (check size of 1° and 0.25°) on a calibrated monitor and flash light stimuli on a MINIganzfeld I8. The patient was placed one meter away from the monitor, the test was performed monocularly, and electrodes were placed on the midline: active (Oz) was placed on the occipital scalp (10% of the nasion-inion distance above the inion), ground was attached to the patient’s forehead, and the reference (Fz) was placed 30% of the nasion-inion distance above the nasion.

The measurements were performed at the beginning of the study (T0), then the patients were given niacinamide capsules (500 mg, commercially available capsules from Health4All, Blackpool, UK) to take one daily for 6 months. After this period, all measurements were performed once again. During this period, the glaucoma treatment had not been modified by the clinician following the patients. Self-reported adherence to niacinamide was 100%, and no patients reported adverse reactions throughout the study period.

### 2.2. Statistical Analysis

Analyses were performed at the eye level, with explicit modeling of within-subject correlation when both eyes from the same participant were included. Exploratory eye-level comparisons were performed using paired-eye data where appropriate, studying categorical and continuous data. Eye-level paired analyses are reported as descriptive and exploratory only, and were not used for primary inference. For categorical variables the absolute and relative frequencies were provided. For continuous variables, the mean ± standard deviation, median (interquartile range) values were calculated, and their normality was checked using the Shapiro–Wilk test. If not normal (Shapiro–Wilk *p*-value less than 0.05), Wilcoxon signed-rank test was used to compare their levels between baseline and after 6 months of B3 supplementation; otherwise, the *t*-test for paired samples was performed. Effect sizes were calculated as rank-biserial correlation with 95% confidence intervals (95% CI) derived from bootstrap procedures. Because of the multiple comparisons (RNFL, GCC, three VEP measures, MD, PSD), Bonferroni (0.05/number of test) corrections were used. For the 14 exploratory comparisons presented in Table 1, statistical significance was assessed against a Bonferroni-adjusted α-level of 0.05/14 = 0.0036, while unadjusted *p*-values are reported.

For each modality, change scores (Δ = T6 − T0) were computed for visual field (ΔMD, ΔPSD), OCT (ΔRNFL, ΔGCC), and VEP (ΔP2, ΔP100_1, ΔP100_15). A heatmap was used to visually illustrate the correlations between variables (Pearson’s correlation coefficient, Pearson’s r, or Spearman correlation coefficient rho, ρ, were calculated). A weak correlation has the coefficient between 0.3 and −0.3, a moderate correlation between 0.3 and 0.5 or between −0.3 and −0.5, and a strong correlation over 0.5 or under −0.5.

Because both eyes from some patients were included, a sensitivity analysis was performed to account for potential inter-eye correlation, whereas the paired-eye analyses had an explicitly secondary and exploratory role. Two complementary approaches were used as the primary analytical approach: Generalized Estimating Equations (GEE) with patient ID as the clustering variable and an exchangeable working correlation matrix, and Linear Mixed-Effects Models (LME) with a random intercept for patient ID to account for between-patient variability. These models were applied to ΔMD to verify whether the primary findings persisted after accounting for within-subject clustering. These clustered models form the basis for primary statistical inference, while paired eye-level analyses are presented as exploratory.

The *p* value of 0.05 was chosen as representing statistical significance. All statistical calculations were performed in Python (version 3.9.12) using the *statsmodels* library.

## 3. Results

### 3.1. Patients’ Characteristics

Fifty-eight patients with glaucoma were evaluated in order to determine the presence of the eligibility criteria and underwent investigations and B3 supplementation. In total, from these patients, 111 eyes were analyzed: 71 (63.96%) eyes with Stage 1 glaucoma, 20 (18.01%) eyes with stage 2, 8 (7.2%) eyes with stage 3, and 12 (10.81%) eyes with stage 4 glaucoma.

The mean ± standard deviation age of the patients was 66.69 ± 11.72 years, the mean ± standard deviation duration of the disease was 8.16 ± 5.06 years, and the mean ± standard deviation visual acuity (LogMAR scale) was 0.31 ± 0.57. Most patients were women (45, 77.6%), as shown in Table 1.

Patients administered an average of 2.31 antiglaucoma molecules (SD 0.98) daily. A total of 20 eyes (18.02%) had undergone glaucoma surgery.

The right vs. left eye baseline characteristics are nearly identical, with no clinically meaningful differences in age, sex, glaucoma stage, OCT parameters, VEP values, or VF results.

### 3.2. Differences in Visual Field Parameters After B3 Supplementation

Both eyes were included, and primary inference was based on clustered analyses accounting for within-subject correlation. Eye-level paired analyses are presented for descriptive comparison only. All eyes were analyzed, comprising 111 pairs of T0-T6 for MD and PSD.

**Figure 1:**
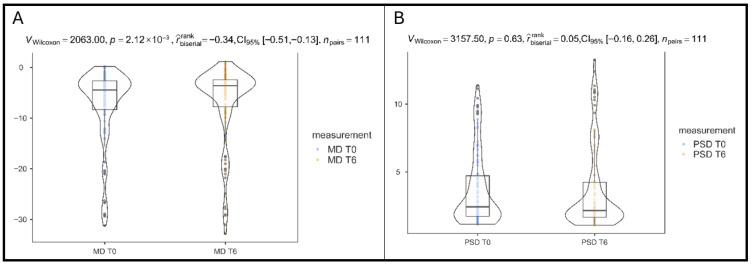
(**A**). Differences in MD after B3 supplementation. (**B**). Differences in PSD after B3 supplementation.

The comparison of MD at baseline and after six months of B3 supplementation showed a modest but statistically significant improvement. Exploratory eye-level paired analyses using a Wilcoxon signed-rank test demonstrated a directional shift toward less negative MD values after 6 months of B3 supplementation (*p* = 0.002, rank-biserial effect size = −0.34, 95% CI: −0.51 to −0.13), as shown in Figure 1A. The effect size indicates a small-to-moderate improvement in global visual field sensitivity from −7.23 ± 7.57 at baseline to −6.79 ± 7.79 at T6, but this finding was not retained after adjustment for within-subject clustering.

In contrast to MD, PSD values did not change significantly over the 6-month period of supplementation (*p* = 0.63, rank-biserial effect size = 0.05, 95% CI: −0.16 to 0.26), as shown in Figure 1B, with changes from 3.72 ± 2.74 at baseline to 3.65 ± 3.08 at T6.

Thus, VF analysis revealed a slight global improvement of function without evidence of new focal damage or improvement.

### 3.3. Differences in OCT Parameters After B3 Supplementation

#### 3.3.1. Differences in Retinal Nerve Fiber Layer After B3 Supplementation

**Figure 2:**
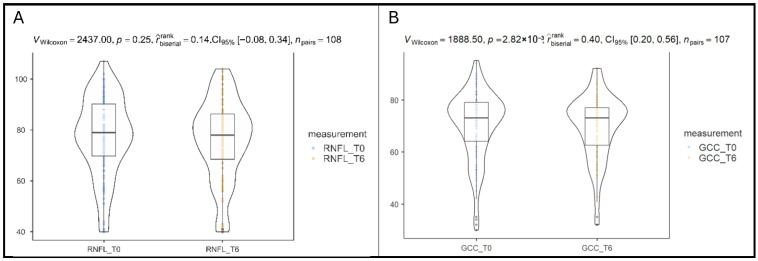
(**A**). Differences in retinal nerve fiber layer after B3 supplementation. (**B**). Differences in ganglion cell complex after B3 supplementation.

Peripapillary RNFL thickness did not show a statistically significant change over the same period (*p* = 0.25, rank-biserial effect size = 0.14, 95% CI: −0.08 to 0.34), as illustrated in Figure 2A. The effect size is small, and the confidence interval includes zero, indicating no consistent thickening or thinning in RNFL across the cohort, changing from 77.2 ± 15.5 to 76.1 ± 15.8. The assessment of the 108 paired measurements reflected structural stability of the optic nerve axonal layer over the 6-month period. In clustered analyses, RNFL thickness showed a small mean decrease (−1.26 µm) that reached statistical significance in the GEE model (*p* = 0.046), although the magnitude was below commonly accepted thresholds for clinically meaningful change.

#### 3.3.2. Differences in Ganglion Cell Complex After B3 Supplementation

A significant decrease in macular ganglion cell complex thickness was observed in paired eye-level analysis after six months of B3 supplementation (*p* = 0.002, rank-biserial effect size = 0.40, 95% CI: 0.20 to 0.56), as illustrated in Figure 2B. The effect size is moderate and negative, indicating that overall GCC thickness decreased from baseline (70.2 ± 13.3) to 6 months (69.6 ± 12.5). However, this change did not remain statistically significant after accounting for inter-eye correlation using clustered models, as shown in Section 3.6.

### 3.4. Differences in VEP Parameters After B3 Supplementation

No VEP latency parameter met statistical significance, although they showed consistent and physiologically meaningful trends toward latency reductions.

#### 3.4.1. Differences in P2 Latency After B3 Supplementation

**Figure 3:**
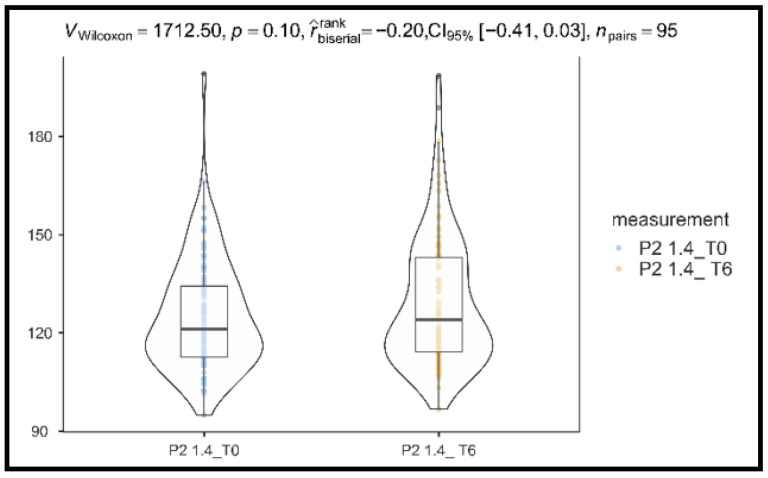
Differences in VEP parameters for P2 latency after B3 supplementation.

The comparison between baseline (125 ± 17.0) and 6 months (129 ± 19.9) showed no statistically significant change in P2 1.4 Hz latency (*p* = 0.10, rank-biserial effect size = −0.20, 95% CI: −0.41 to 0.03), as shown in Figure 3.

#### 3.4.2. Differences in P100 Latency After B3 Supplementation

**Figure 4:**
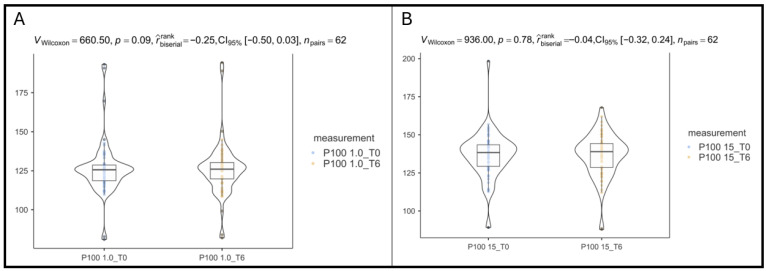
Differences in VEP parameters for P100 latency after B3 supplementation: (**A**). At 1 min stimulation. (**B**). At 15 s stimulation.

For the larger stimuli (1-degree arc) P100 latency, there is a non-significant tendency toward lower latencies after B3 supplementation (from 126 ± 16.8 at baseline to 124 ± 15.6 at T6), with a small-to-moderate effect size but wide confidence intervals (*p* = 0.09, rank-biserial effect size = −0.25, 95% CI: −0.50 to 0.03), as shown in Figure 4A.

For the smaller stimuli (15 min arc) P100 latency, no meaningful change was observed in this parameter (from 136 ± 15.4 baseline to 136 ± 13.7 at T6); effect size is essentially null (*p* = 0.78, rank-biserial effect size = −0.04, 95% CI: −0.32 to 0.24), as shown in Figure 4B.

Taken together, VEP results indicate directional but subthreshold improvement in central/parvocellular processing.

### 3.5. Structure–Function Relationships

**Figure 5:**
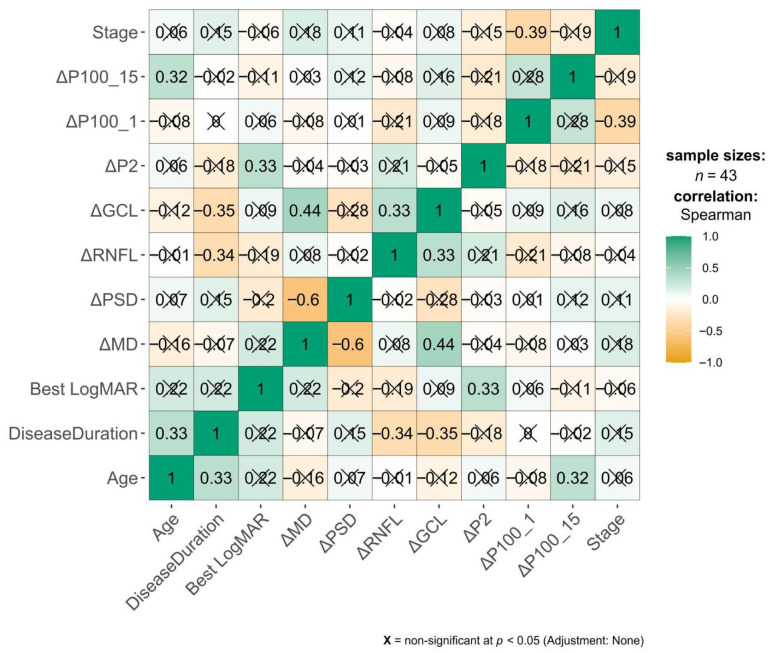
Heatmap with Spearman correlation matrix.

The change-change Spearman correlation analysis revealed several meaningful associations across functional (VF), structural (OCT), and electrophysiological (VEP) domains, as shown in the correlation heatmap in Figure 5. Smaller check-size (15 min) P100 latency showed no meaningful change or correlation.

Regarding the visual fields and structure, ΔMD strongly correlated with ΔPSD (ρ = –0.6, *p* = 0.001). Put simply, there was a negative correlation—as ΔMD is more positive (the MD after treatment is higher, less negative, closer to 0—less global VF deviation), the ΔPSD is more negative (the PSD after treatment is smaller, closer to 0—less focused VF defects). This reflects the expected diffuse-versus-focal relationships in VF metrics. ΔMD correlated significantly with ΔGCC (ρ = 0.44), but not with ΔRNFL (ρ = 0.08). As a positive correlation, a higher, less negative MD after treatment is correlated with a higher GCC thickness after treatment—reflecting a good concordance between VF function and central macular structure.

Regarding intra-OCT relationships, ΔGCC correlated positively with ΔRNFL (ρ = 0.33), suggesting coordinated morphology of macular and peripapillary inner retinal layers.

Associations between ΔRNFL and VEP parameters were weak and non-significant.

Regarding VEP internal relationships, ΔP100 15′ non-significant correlated with ΔP100 1° (ρ = 0.28), and neither correlated meaningfully with VF or OCT changes.

The glaucoma stage showed a moderate negative correlation with ΔP100 (ρ = −0.39), indicating that more advanced disease was associated with smaller improvements (or greater worsening) in P100 latency. This association was among the strongest observed for the stage across the analyzed outcomes. Correlations between the stage and other VEP or structural change metrics were weak (|ρ| < 0.2) and not statistically significant.

### 3.6. Clustered Analyses Accounting for Inter-Eye Correlation—Primary Clustered Analysis

Clustered analyses using GEE and linear mixed-effects models were performed as the primary analytical approach to account for within-subject correlation due to bilateral eye inclusion. Because both eyes were included, sensitivity analyses using linear mixed-effects models with patient ID as a random effect were performed to account for within-subject correlation.

The GEE model estimated a mean ΔMD of 0.43 dB (coeff = 0.426, SE = 0.258), which did not reach statistical significance (z = 1.65, *p* = 0.099, 95% CI: –0.08 to 0.93). The linear mixed-effects model yielded an almost identical estimate (0.43 dB, SE = 0.261), also non-significant (z = 1.64, *p* = 0.101, 95% CI: –0.08 to 0.94), with a between-patient variance of 2.51 dB^2^.

These primary clustered models demonstrate that, once inter-eye correlation is explicitly addressed, the observed changes are small in magnitude and do not provide evidence of a clinically meaningful functional or structural effect. Importantly, both methods produced ΔMD estimates in the same direction and magnitude as the unadjusted non-parametric analysis, indicating that the overall conclusion—slight, statistically insignificant improvement—remains unchanged.

On average, RNFL thickness decreased by about 1.3 µm over 6 months, and this was statistically significant in the GEE model (*p* = 0.046). GCC did not show a statistically significant change when inter-eye correlation is accounted for (*p* = 0.54), meaning that after adjusting for clustering and missing data, there is no robust evidence of a systematic GCC increase or decrease in this model.

These models confirmed the direction and magnitude of almost all primary findings. After properly adjusting for inter-eye correlation, most parameters are stable over 6 months; the statistically significant signals (ΔRNFL: GEE coef = −1.26 μm, *p* = 0.046, and ΔP2: GEE coef = 3.9 ms, *p* = 0.039) are small in magnitude.

## 4. Discussion

This multimodal analysis provides an integrated evaluation of the structural, functional, and electrophysiological effects of six months of niacinamide supplementation in POAG. While exploratory eye-level analyses suggested modest changes, clustered analyses accounting for inter-eye correlation indicate overall functional and structural stability rather than definitive improvement. When accounting for inter-eye similarities in patients whose both eyes were analyzed, the MD improvement lost the statistical significance; however, a small worsening in RNFL and P2 latency became statistically significant. This finding underscores the importance of accounting for inter-eye correlation in ophthalmic studies, as unadjusted eye-level analyses may overestimate statistical significance. Lastly, the variations before and after treatment showed correlations between them: the MD improvement correlated in a statistically significant manner with both a GCC increase and a PSD decrease. Overall, the improvements are modest, and the regressions were also of small magnitude, collectively indicating overall stability rather than progression or consistent improvement.

Niacinamide in glaucoma is a promising neuroprotective treatment, with robust data in preclinical studies of animal models [14]. While many clinical trials are registered, the data that has been published so far suggest that, similarly to our data, the role of niacinamide in glaucoma is complicated and has still not clearly shown its benefit [14].

The analysis of perimetry evolution has been shown to be complex: while the initial Wilcoxon test suggested a significant MD improvement, the cluster-adjusted models demonstrated that the mean ΔMD (~+0.4 dB) did not reach statistical significance. This variance is well within the expected test–retest variability: a large study involving stable POAG eyes reveals a mean test–retest MD difference of 0.7 dB [22]. In parallel, PSD remained unchanged, confirming stable localized defect patterns and absence of focal scotoma evolution [16].

Similarly, no significant alterations of mean PSD and MD were observed in a randomized controlled trial in which patients received either 1.5 g/day or 3 g/day of niacinamide [23] or one involving normal tension glaucoma patients receiving 1 g/day and 2 g/day of placebo or niacinamide for 12 weeks [24]. A phase 2 placebo-controlled trial which also administered niacinamide and pyruvate had followed patients using automated perimetry: similarly to our study, it had not identified a quicker MD deterioration in the study group compared to the placebo group; however, it detected a higher rate of PSD improvement and significantly more VF tested points that improved [25]. Overall, it detected a rate of MD change of 0.04 dB/week, which would approximate to 0.96 dB/6 months, compared to the 0.43 dB detected in our cohort [25]. It is important for future studies to detect the potential added value of the pyruvate administered in tandem with the niacinamide.

Electrophysiologically, P100 responses remained stable at both spatial frequencies, underscoring preserved conduction along parvocellular and magnocellular pathways. The modest increase in P2 latency (+3.9 ms, *p* = 0.039) reached statistical significance in the GEE model but was small and must be viewed within the context of normal intra-individual VEP variability, which was calculated by other studies in healthy subjects to span up to 9–11 ms [26]. There was no consistent alignment between VEP and OCT changes, further suggesting that the detected P2 shift likely reflects test variability rather than meaningful neurophysiological deterioration.

It is notable from the literature that, while there are few clinical trials involving niacinamide in glaucoma, those which detected statistically significant improvements were those that did so using electroretinography [23,24]—supporting the intracellular metabolic benefit of niacinamide, more so than the larger structural or perimetric benefit expected when measuring visual field or OCT parameters.

Structural OCT findings were likewise consistent with stability. GCC thickness did not demonstrate a significant change in the cluster-adjusted model, and RNFL thinning detected by the same model (≈1.3 µm) was statistically significant but small in magnitude and compatible with physiologic fluctuation and measurement variability. Similarly to our study, the previously mentioned clinical trials had not detected any RNFL improvements after 12 weeks of niacinamide + pyruvate oral supplementation [25] or after 12 weeks of crossover placebo and niacinamide (1.5 g/day or 3 g/day) [23].

It is known that OCT markers, as they assess the retinal structures affected by IOP, vary in line with the glaucomatous progression [27]. Repeatability tests of OCT devices in healthy eyes reveal an Overall Within-Subject standard deviation of RNFL of 1.11 µm [28]. The seminal Framingham study also investigated the intraindividual variability of RNFL: it revealed that, on average, any observed change in RNFL thickness exceeding 4.47 μm may be considered meaningful and not attributable to chance within a measurement session [29]. Overall, studies suggest that a reasonable threshold for glaucomatous progression could be a difference of 3–4 µm [28,29], which was not achieved by our study, therefore not supporting the idea that the RNFL thinning detected mirrors true glaucomatous degradation of the optic nerve.

The moderate association between ΔGCC and ΔMD (ρ = 0.44) indicates that eyes showing greater macular stability or minor improvement in GCC tended to maintain better global VF sensitivity. No other correlations reached significance, emphasizing the independence of short-term fluctuations across OCT, VEP, and VF modalities. This correlation between the VF parameters variability and OCT parameters variability is known; moreover, the correlation is known to be stronger to GCC than to RNFL, similar to our results [30].

The observed inverse relationship between the glaucoma stage and ΔP100 (rho = −0.39) underlines that a potential neuroprotective effect of niacinamide is more evident in earlier disease stages, particularly stage 1. P100 latency reflects functional integrity of the visual pathway [31]; therefore, greater responsiveness in early-stage glaucoma is biologically plausible, as retinal ganglion cells and downstream pathways are more likely to retain metabolic reserve and functional plasticity [32]. In contrast, advanced stages are characterized by irreversible axonal loss and reduced capacity for functional recovery [33], which likely attenuates measurable electrophysiological benefit despite supplementation. This observation suggests that niacinamide may be most useful administered in early glaucoma cases, and this different behavior of glaucoma severities should inform the stratification of future studies.

Several limitations were present in this study: the follow-up was relatively short, of 6 months, and a longer follow-up may be more suitable to pick up subtle improvements in glaucoma progression or improvement. This study was also developed to generate hypotheses and estimate variance for a future RCT. Our 6-month data represents an interim analysis to assess acute, short-term responses, safety, and to justify the continuation or design of the longer, registered one-year trial. Moreover, our exploratory study is limited by the lack of a control group to clearly identify the effects of the niacinamide intervention, and a larger number of patients would allow for a better understanding of B3 supplementation and its effects depending on glaucoma severity and stage. In terms of the intervention, while other glaucoma trials involved higher doses of niacinamide administered [23,24,25], in our study we chose a conservative, safety-oriented dose appropriate for an exploratory, first-phase clinical study in a real-world glaucoma population, chosen to minimize the risk of adverse effects in an older cohort with comorbidities and to assess feasibility and short-term safety before escalating dosing in future randomized trials.

Moreover, the patients were not blinded to the intervention, which may have an impact, particularly when evaluating subjective measures, such as quality of life [19] or when undergoing focus-dependent tests, such as automated perimetry. Also, more detailed segmentation of the data may reveal important differences: it is known that superior, inferior, nasal, and temporal peripapillary RNFL have different dynamics in glaucomatous progression, which may not be obvious by analyzing the global thickness only [28]. Last but not least, the serum concentration of niacinamide and its metabolites was not assessed, nor was the daily intake from the diet, with a higher intake being known to be associated with lower prevalence of glaucoma [12]. All in all, our study presents two distinct advantages: the inclusion of visual evoked potential measurements, along with OCT and perimetry, and the longer duration of B3 administration (6 months, compared to 12 weeks in the previously published trials) [23,24,25].

Considered together, the results demonstrate that niacinamide supplementation was not associated with detectable short-term optic nerve degradation; however, neither definitive structural or electrophysiological improvement was detected.

## 5. Conclusions

Six months of niacinamide supplementation was associated with overall functional, structural, and electrophysiological stability when inter-eye correlation was appropriately modeled. No clinically meaningful improvement or deterioration was detected over the study period. Visual fields showed no clinically meaningful progression, OCT metrics remained largely unchanged aside from a small RNFL fluctuation of uncertain relevance, and VEP responses were predominantly stable with only minor variability in P2 latency. It highlights the need for larger, randomized, placebo-controlled trials to determine neuroprotective efficacy.

## Figures and Tables

**Table 1:** Characteristics of the included patients.

Characteristics	Right Eye (N = 58)	Left Eye (N = 53)	*p*-Value
Age Mean ± SD Median (Interquartile Range) Shapiro–Wilk *p*	66.7 ± 11.7 69 (16.8) 0.032	66.6 ± 11.8 69 (17) 0.035	
Sex Female (%) Male (%)	45 (77.6%) 13 (22.4%)	42 (79.2%) 11 (20.8%)	
Baseline glaucoma severity Stage 1 (%) Stage 2 (%) Stage 3 (%) Stage 4 (%)	31 (53.4%) 12 (20.7%) 8 (13.8%) 7 (12.1%)	26 (49.1%) 12 (22.6%) 8 (15.1%) 7 (13.2%)	
C/D Mean ± SD Median (Interquartile Range) Shapiro–Wilk *p*	0.59 ± 0.19 0.57 (0.3) 0.521	0.57 ± 0.21 0.6 (0.29) 0.394	
IOP baseline Mean ± SD Median (Interquartile Range) Shapiro–Wilk *p*	15.5 ± 2.8 15 (4.5) 0.009	16.2 ± 2.62 16 (4) 0.052	
IOP T6 Mean ± SD Median (Interquartile Range) Shapiro–Wilk *p*	15.2 ± 3.1 15 (6) 0.005	15.7 ± 2.83 15 (5) 0.026	
Optic Coherence Tomography (OCT)			
RNFL baseline Mean ± SD Median (Interquartile Range) Shapiro–Wilk *p*	77.6 ±15.7 77 (22) 0.026	76.7 ± 15.5 79 (20.5) 0.233	0.707
RNFL T6 Mean ± SD Median (Interquartile Range) Shapiro–Wilk *p*	77.1 ± 16.1 78 (19) 0.109	75 ± 15.4 77.5 (17.8) 0.02	0.567
GCC baseline Mean ± SD Median (Interquartile Range) Shapiro–Wilk *p*	70 ± 12.8 72.5 (16) 0.002	70.5 ± 13.9 75 (15.5) 0.002	0.637
GCC T6 Mean ± SD Median (Interquartile Range) Shapiro–Wilk *p*	69.1 ± 12.4 73.5 (15.5) 0.005	70.1 ± 12.7 73 (12.5) 0.008	0.680
VEP parameters			
P2 1.4 Hz baseline Mean ± SD Median (Interquartile Range) Shapiro–Wilk *p*	126 ± 18.5 122 (23.9) <0.001	124 ± 15.4 120 (19.9) 0.173	0.774
P2 1.4 Hz T6 Mean ± SD Median (Interquartile Range) Shapiro–Wilk *p*	130 ± 20.1 125 (28.7) <0.001	128 ± 19.9 121 (26.3) 0.007	0.617
P100 1.0 baseline Mean ± SD Median (Interquartile Range) Shapiro–Wilk *p*	126 ± 15.6 126 (8.98) <0.001	127 ± 18.2 125 (10.1) <0.001	0.715
P100 1.0 T6 Mean ± SD Median (Interquartile Range) Shapiro–Wilk *p*	125 ± 15.1 126 (11.3) <0.001	123 ±16.2 122 (15.8) <0.001	0.435
P100 15 baseline Mean ± SD Median (Interquartile Range) Shapiro–Wilk *p*	136 ± 14.1 138 (14.7) 0.018	137 ± 16.9 138 (12.4) <0.001	0.739
P100 15 T6 Mean ± SD Median (Interquartile Range) Shapiro–Wilk *p*	137 ± 13.9 139 (14.9) 0.062	134 ± 13.5 136 (12.9) 0.086	0.384
Visual Field			
MD (db, decibels) baseline Mean ± SD Median (Interquartile Range) Shapiro–Wilk *p*	−7.03 ± 7.62 −4.48 (5.7) <0.001	−7.46 ± 7.59 −4.38 (5.94) <0.001	0.797
MD (db, decibels) T6 Mean ± SD Median (Interquartile Range) Shapiro–Wilk *p*	−6.56 ± 7.59 −3.69 (4.57) <0.001	−7.03 ± 8.08 −3.61 (6.09) <0.001	0.885
PSD baseline Mean ± SD Median (Interquartile Range) Shapiro–Wilk *p*	3.53 ± 2.62 2.30 (2.54) <0.001	3.93 ± 2.88 2.48 (4.18) <0.001	0.499
PSD T6 Mean ± SD Median (Interquartile Range) Shapiro–Wilk *p*	3.53 ± 2.99 2.12 (2.4) <0.001	3.78 ± 3.19 2.28 (3.08) <0.001	0.890

RNFL, retinal nerve fiber layer; GCC, ganglion cell complex; MD, mean deviation; PSD, Pattern Standard Deviation. *p*-values shown are unadjusted (raw) *p*-values. For the 14 comparisons in this table, statistical significance was evaluated using a Bonferroni-adjusted threshold of α = 0.0036 (0.05/14). Sample sizes (number of eyes) contributing to each comparison are reported within the table.

## Data Availability

The data sets generated during and/or analyzed during the current study are available from the corresponding author on reasonable request.

## Abbreviations

The following abbreviations are used in this manuscript:

HFA	Humphrey Field Analyzer
IOP	Intraocular pressure
MD	Mean Deviation
OCT	Optical coherence tomography
ONH	Optic Nerve Head
POAG	Primary open-angle glaucoma
PSD	Pattern Standard Deviation
PV	Peripheral vision
VEP	Visually evoked potential
VF	Visual field
IPL	Inner plexiform Layer
GCC	Ganglion Cell Complex
GCL	Ganglion Cell Layer
NFL	Retinal nerve fiber layer
PRVEP	Pattern-reversal visually evoked potential
